# Treatment selection for patients with relapsed or refractory follicular lymphoma

**DOI:** 10.3389/fonc.2023.1120358

**Published:** 2023-03-07

**Authors:** Alan Z. Skarbnik, Krish Patel

**Affiliations:** ^1^ Novant Health Lymphoma and CLL Program, Charlotte, NC, United States; ^2^ Swedish Cancer Institute, Center for Blood Disorders and Cellular Therapy, Seattle, WA, United States

**Keywords:** B-cell lymphoma, bispecific antibodies, CD19 antigen, copanlisib, EZH2 inhibitor, lymphoma, tazemetostat

## Abstract

Follicular lymphoma (FL) is the second most common lymphoma in the United States and is characterized by a variable clinical course, disease heterogeneity, and a relapse-and-remittance pattern historically accompanied by successive shortening of clinical response with every line of treatment. Factors such as progression of disease within 24 months of initial treatment are associated with poor survival outcomes. Although rituximab-based regimens are preferred for early lines of treatment, no clear standard of care exists for treatment of FL in the third-line setting or later as approved third-line treatments have not been compared in a prospective, randomized clinical trial. Rather, physicians may choose from several therapeutic classes with different safety profiles and dosing regimens, with consideration of patient and disease factors. Here we describe 2 hypothetical patients with relapsing or remitting FL, an elderly patient with comorbidities, and a younger patient whose FL progressed within 24 months. These cases are used to highlight key factors that clinicians should consider when selecting therapies for relapsed or refractory FL, such as patient frailty, age, comorbidities, as well as quality of life and patient-specific preferences for less intrusive treatment regimens or longer remission times.

## 1 Introduction

Follicular lymphoma (FL) is the second-most common subtype of lymphoma, accounting for approximately 20% of all cases of non-Hodgkin lymphoma in the United States and Europe (1). Although FL is generally considered an indolent subtype of lymphoma, there is significant clinical and biological heterogeneity in its presentation (2). Most patients present with advanced-stage disease (stage 3/4) (3) that is considered incurable. Median age at diagnosis is 63 years, and FL carries a 14-year median overall survival (OS), with more than 90% of patients surviving beyond 5 years of initial diagnosis (3–5).

Given its incurability and generally indolent behavior (5), most patients with FL initially may be clinically observed until or unless symptoms or burden of disease demand initiation of therapy. Frontline therapy for FL usually consists of a combination of an anti-CD20 monoclonal antibody and chemotherapy (6, 7); the most-used regimen in the United States is a combination of bendamustine and rituximab (BR) (8). As reported in the initial phase 3 BRIGHT study (NCT00877006) (9), this regimen lead to an objective response rate (ORR) of 97% and a complete response (CR) rate of 31%; progression-free survival (PFS) at 5 years follow-up was 65.5% (8). For frail patients who may not tolerate chemoimmunotherapy, rituximab as a single agent or in combination with lenalidomide are alternative treatment options (6, 10). In the RELEVANCE trial (NCT01476787 and NCT01650701), lenalidomide combined with rituximab for 18 cycles of 28 days, followed by 6 additional doses of maintenance rituximab every 8 weeks, yielded a 48% CR rate and 77% PFS at 3 years and was associated with lower rates of high-grade neutropenia and febrile neutropenia than chemoimmunotherapy when given in a frontline setting (11).

Most patients with FL will eventually present with relapsed disease (5), and treatment options in this setting depend highly on performance status, disease burden, potential presence of high-grade transformation, and duration of response (DOR) after frontline therapy (6, 7, 10). For patients who received chemoimmunotherapy as frontline treatment for FL, progression of disease within 24 months of therapy initiation (POD24) is associated with poorer outcomes (5-year OS, 73.5% vs 95.4% for non-POD24) (12). POD24 occurs in approximately 20% of patients after front-line chemoimmunotherapy (13), and management options for such patients often include consideration of clinical trials, anthracycline-based chemotherapy (if not previously received), or platinum chemoimmunotherapy followed by autologous stem cell transplantation (ASCT). In a subgroup analysis of the AUGMENT study (NCT01938001), lenalidomide and rituximab demonstrated median PFS of 30.4 months in patients with POD24 and this combination may be a consideration for some patients (14). Recent data also suggest that chimeric antigen receptor (CAR) T-cell therapy is effective for patients experiencing POD24 (15), although currently, such therapy is typically available to patients after 2 or more lines of therapy (16). For patients not fit for intensive treatment approaches at relapse, lenalidomide plus rituximab is a commonly chosen treatment approach (17).

Historically, DOR tends to be shorter after each line of treatment for FL (18, 19), creating additional challenges when selecting therapies in the third line or later. There are multiple available therapies in this setting but no single standard of care (6, 7, 10). The lack of prospective, randomized phase 3 studies comparing recently approved options for third-line or later treatment leads to physicians choosing treatments based on single-arm trial data, largely relying on response rates, safety profiles, accessibility of therapy, and patient preferences. In this article, we discuss potential approaches for patients with relapsed/refractory (R/R) FL in the third-line setting and beyond, illustrated with 2 hypothetical patient cases.

## 2 Hypothetical case scenarios

### 2.1 Patient 1

A 76-year-old man was diagnosed in 2013 with grade 1–2 stage 3 FL. The presence of bulky lymphadenopathy (>7 cm), fatigue, and abdominal pain led to the initiation of therapy with BR; the patient achieved CR after 6 cycles of therapy, confirmed by positron emission tomography (PET). In 2019, the patient presented with recurrence of disease, with diffuse, symptomatic adenopathy. At this time, his FL was treated with lenalidomide plus rituximab for 12 cycles, achieving a partial response to therapy. Within 6 months of therapy completion, the patient presented with new cervical and inguinal lymphadenopathy indicative of disease progression. This patient had multiple comorbidities, including hypertension, atrial fibrillation, hypothyroidism, and stage 3 chronic kidney disease. He presented with an Eastern Cooperative Oncology Group performance status (ECOG PS) of 1 and was relatively active, playing golf 3 to 4 times per week. This patient lived far from his oncologist’s treatment center and placed significant importance on minimizing health care visits. He was offered treatment with copanlisib, an intravenously administered phosphoinositide 3 (PI3)-kinase inhibitor approved for the treatment of FL, versus tazemetostat, an oral inhibitor of enhancer of zeste homolog 2 (EZH2), also approved for the treatment of R/R FL. Presented with both options, the patient chose oral tazemetostat therapy, which would minimize the number of his office visits.

### 2.2 Patient 2

A 55-year-old woman was diagnosed in 2017 with grade 3a stage 4 FL, presenting with anemia, thrombocytopenia, abdominal pain, and splenomegaly. She was treated with 6 cycles of BR and achieved CR confirmed by a PET scan. Within 20 months of initiation of therapy, the patient presented with progressive fatigue, anemia, right upper quadrant fullness, and early satiety. A PET scan revealed multistation hypermetabolic adenopathy, suggestive of a recurrence of FL. A biopsy of a representative lymph node confirmed relapsed FL without high-grade transformation. This patient was treated with 12 cycles of lenalidomide and obinutuzumab, achieving CR confirmed by a PET scan. However, within 6 months of therapy completion, she presented again with anemia, progressive adenopathy, and splenomegaly. A repeat biopsy confirmed recurrent grade 3a FL. She was otherwise healthy, with an ECOG PS of 0. The patient was given several treatment options, including CAR T-cell therapy, chemotherapy salvage followed by ASCT, copanlisib, and tazemetostat. Due to her working considerations, she preferred a short treatment course with the potential for prolonged remission. As such, she and her physician decided on CAR T-cell therapy, given as a single treatment infusion.

## 3 Discussion

Selection of treatment for FL after multiple lines of therapy remains challenging for both physicians and patients. As relapsed FL is an incurable disease, treatment efficacy should be balanced with toxicity and preservation of quality of life for patients (13). In this paper, we described 2 hypothetical patient case scenarios to better understand potential treatment options for patients with R/R FL in the third-line setting and beyond. Currently, several approved therapies are available for third-line or later treatment of FL (6, 7, 10), but they have to date not been compared with each other in prospective, randomized, controlled clinical trials.

Copanlisib, an inhibitor of phosphoinositide 3-kinase (PI3K) alpha and delta isoforms, is indicated in the United States for the treatment of adult patients with relapsed FL who have received at least 2 prior systemic therapies (20). It is given intravenously on days 1, 8, and 15 of 28-day cycles until disease progression or unacceptable toxicity. The CHRONOS-1 study (NCT01660451) (21) included 104 patients with FL requiring treatment (≥3rd line) who received copanlisib as a single agent (**Table 1**), yielding an ORR of 60.6%, median DOR of 14.1 months, PFS of 12.5 months, and OS of 42.6 months (27). Treatment-emergent adverse events (TEAEs) included hyperglycemia (50%; 33.1% grade ≥3), diarrhea (35.2%, 8.5% grade ≥3), hypertension (29.6%; 23.9% grade ≥3), neutropenia (28.9%; 24% grade ≥3), and pneumonitis (6.3%; 1.4% grade ≥3) (27). More recently, copanlisib was given together with rituximab in the phase 3, randomized, controlled CHRONOS-3 trial (NCT02367040; **Table 1**), yielding a median PFS of 21.5 months vs 13.8 months for rituximab plus placebo (22).

**Table 1:** Patient populations in B-cell lymphoma clinical trials.

Clinical Trial Name (clinicaltrials.gov identifier)	Interventional Arms, n	Median Age (range), y	Median Prior Lines of Therapy, n (range)	POD24, %	Refractory to Last Treatment, %	Refractory to Rituximab, %
CHRONOS-1 (NCT01660451) (21)	Copanlisib, N=142 (104 FL)	63 (25–82)	3 (2─9)	NA	61	56
CHRONOS-3 (NCT02367040) (22)	Copanlisib plus rituximab, n=307 (184 FL)	63 (54─70)	2 (1─3)	NA	NA	NA
	Rituximab plus placebo, n=151 (91 FL)	62 (53─70)	2 (1─NA)	NA	NA	NA
ZUMA-5 (NCT03105336) (15)	Axi-cel, N=146 (124 FL)	61 (34─79)	3 (1─10)	55	68	NA
E7438-G000-101 (NCT01897571) (23)	Tazemetostat, n=45 (45 FL)^a^	62 (57─68)	2 (2─3)	42	49	49
	Tazemetostat, n=54 (54 FL)^b^	61 (53─67)	3 (2─5)	59	41	59
GO29781 (NCT02500407) (24, 25)	Mosunetuzumab, N=218 (62 FL)	59 (27─85)	3 (2─11)	48	NA	53^c^
GO40516 (NCT03671018) (26)	Mosunetuzumab and polatuzumab vedotin, N=22 (10 FL)	70 (38-81)	3 (1-10)	NA	77	86^c^

a Patients with MT *EZH2* FL.

b Patients with WT *EZH2* FL.

c Refractory to last prior anti-CD20 therapy.

EZH2, enhancer of zeste homolog 2; FL, follicular lymphoma; MT, mutant; NA, not available; POD24, progression of disease within 24 months, WT, wild-type.

Axicabtagene ciloleucel (axi-cel) is a CD19-directed autologous CAR T-cell therapy approved in the United States and Europe for third-line or later treatment of R/R FL (16, 28). The ZUMA-5 study (NCT03105336) was a single-arm, phase 2 trial evaluating axi-cel in R/R FL or marginal zone lymphoma (29). Enrolled patients had a median 3 prior lines of therapy (**Table 1**) (15). This study included 124 patients with FL, yielding an ORR of 94% and a CR rate of 79%. Median DOR was 38.6 months at a median of 30.9 months of follow-up in patients with FL (29). Despite the high response rates, CAR T-cell therapy can be delivered only in an accredited treatment center, may require hospitalization, and can lead to both acute (cytokine release syndrome [CRS], neurotoxicity [both reversible with appropriate therapy]) and long-term (cytopenias, hypogammaglobulinemia) toxicities (30). Access to CAR T-cell therapy can be a limiting factor, as patients who live a long distance from a center with this therapy available and those whose psychosocial support is limited may not be able to receive CAR T-cell therapy, despite being medically eligible (30).

Tazemetostat, an oral inhibitor of EZH2, is approved in the United States for adult patients with R/R FL whose tumors are positive for an *EZH2* mutation and who have received at least 2 previous systemic lines of therapy, or in patients with R/R FL who have no satisfactory alternative treatment options (31). EZH2 is an epigenetic regulator with an important role in B-cell maturation. Activating mutations in *EZH2* occur in approximately 20% of patients with FL (23). In a single-arm phase 2 trial (NCT01897571), tazemetostat was given orally twice per day until disease progression or unacceptable toxicity (23). This study included patients with mutant (n=45) or wild-type (n=54) *EZH2* (**Table 1**). For the *EZH2*-mutant cohort, ORR was 69%, median DOR was 10.9 months, and median PFS was 13.8 months. For the *EZH2*-wild type cohort, ORR was 35%, median DOR was 13.0 months, and median PFS was 11.1 months. Grade 3 or higher TEAEs were rare and included anemia (5%), thrombocytopenia (5%), and neutropenia (4%) (23).

The use of CD3 x CD20 bispecific antibodies, such as mosunetuzumab and glofitamab, for treatment of R/R FL is of particular interest owing to preliminary efficacy and DOR data. In a phase 1b/2 study (NCT02500407) (24, 25), patients with R/R B-cell lymphoma received mosunetuzumab monotherapy for up to 17 cycles. After a median of 14.4 months on study, the ORR was 68%, median DOR was 20.4 months, and the median PFS was 11.8 months in patients with FL (25). Grade 3 or higher TEAEs of hypophosphatemia and neutropenia were reported in 23% and 21% of patients, respectively. CRS occurred in 14 patients (23%), and was mostly grade 1 or 2 (n=13). Mosunetuzumab is also being evaluated in a phase 1b/2 study (NCT03671018) in combination with polatuzumab vedotin, a CD79b antibody–drug conjugate, in patients with R/R B-cell lymphoma. For patients with FL (median 3 prior lines of therapy), ORR is 100% (n=3) at median of 9.6 months’ follow-up (26). Mosunetuzumab gained regulatory authorization from the European Medicines Agency and US Food and Drug Administration in 2022 for the treatment of adult patients with R/R FL after at least 2 lines of prior treatment (32, 33). Glofitamab, a 2:1 format CD20 x CD3 bispecific antibody, also demonstrated an 81% ORR and a 70% complete metabolic response in a cohort of patients with grades 1 through 3a FL who had received at least 1 prior therapy (34). The most common adverse event was CRS, occurring in 66% of patients, with most instances being grade 1 or 2 (34). While the initial data for bispecific antibodies in FL are promising, none are currently approved and median follow-up remains short.

Revisiting the cases presented earlier, differing patient factors led one patient to decide to receive tazemetostat while the other chose CAR T-cell therapy. Among therapy options for R/R FL, tazemetostat appears to have a low toxicity burden, with few high-grade AEs and with the potential for durable benefit, with a median PFS rate of approximately 11 to 14 months in third-line or later therapy. Oral dosing without the need for infusion visits may also be desirable for some patients. Among patients who prioritize a fixed duration of treatment and prolonged disease control without continuous therapy, CAR T-cell therapy may be preferred. In addition, the activity of CAR T-cell therapy in patients with biologically adverse risk factors, such as POD24, may also influence its use. However, CAR T-cell therapy can lead to serious toxicities, including high-grade CRS and neurologic toxicity, as well as prolonged cytopenias. Thus, it may be best considered in fit patients with anticipated physiologic reserve to endure typical CAR T-cell therapy–related adverse events. Also, CAR T-cell therapy typically requires that a patient travel to a cellular therapy center and requires appropriate caregiver support. Patients and their physicians should be well informed of the eligibility requirements for CAR T-cell therapy at local cellular therapy centers.

Prospective trials comparing current treatment options are highly needed in this arena, as they would best inform therapeutic choices. In the meantime, clinicians must rely on single-arm data and weigh efficacy, impact on quality of life, and cost of care when selecting a treatment approach for patients with FL who need 3rd-line or beyond treatment.

## Data availability statement

The original contributions presented in the study are included in the article/supplementary material. Further inquiries can be directed to the corresponding author.

## Author contributions

All authors had full access to the patients’ data and contributed to the drafting, critical review, and revision of the manuscript, with the support of a medical writer provided by Epizyme, Inc. All authors contributed to the article and approved the submitted version.
